# A multicenter noninferior randomized controlled study comparing the efficacy of laparoscopic versus abdominal radical hysterectomy for cervical cancer (stage IA1 with LVSI, IA2): study protocol of the LAUNCH 1 trial

**DOI:** 10.1186/s12885-022-09494-4

**Published:** 2022-04-09

**Authors:** Xin Wu, Hao Feng, Shujun Gao, Hua Feng, Wenjing Diao, Hongwei Zhang, Ming Du, Weihua Lou, Xipeng Wang, Tao Zhu, Yuyang Zhang, Weiguo Hu, Xiaohong Xue, Zhiling Zhu, Libing Xiang, Jiarui Li, Xuhong Fang, Yongrui Bai, Yanli Hou, Weili Yan, Ling Qiu, Hailin Yu, Shurong Zhu, Yan Du, Hua Jiang

**Affiliations:** 1 Obstetrics and Gynecology Hospital, Fudan University, Shanghai, 200011 China; 2 Shanghai Jiao Tong University School of Medicine, Renji Hospital, Shanghai, 200127 China; 3 Xinhua Hospital Affiliated to Shanghai Jiao Tong University School of Medicine, Shanghai, 200092 China; 4 Cancer Hospital of the University of Chinese Academy of Sciences (Zhejiang Cancer Hospital), Hangzhou, 310005 China; 5 The First Affiliated Hospital of Wenzhou Medical University, Wenzhou, 325000 China; 6 Zhongshan Hospital Affiliated to Fudan University, Shanghai, 200032 China; 7 Children’s Hospital of Fudan University, Shanghai, 201102 China

**Keywords:** Cervical cancer, Stage IA1 with LVSI and IA2, Laparoscopic radical hysterectomy, Abdominal radical hysterectomy, Progression-free survival, Overall survival, Prognosis

## Abstract

**Background:**

A retrospective study and a randomized controlled trial published in a high quality journal in late 2018 have shown that laparoscopic radical hysterectomy (RH) was associated with worse survival than abdominal RH among patients with early stage cervical cancer. Radical hysterectomy in cervical cancer has been a classic landmark surgery in gynecology, therefore this conclusion is pivotal. The current trial is designed to reconfirm whether there is a difference between laparoscopic RH and abdominal RH in cervical cancer (stage IA1 with LVSI, IA2) patient survival under stringent operation standards and consistent tumor-free technique. This paper reports the rationale, design, and implementation of the trial.

**Methods:**

This is an investigator-initiated, prospective, randomized, open, blinded endpoint (PROBE) controlled trial. A total of 690 patients with stage IA1 (with intravascular), and IA2 cervical cancer will be enrolled over a period of three years. Patients are randomized (1:1) to either the laparoscopic RH or the abdominal RH group. Patients will then be followed-up for at least five years. The primary endpoint will be 5-year progression-free survival. Secondary endpoints will include 5-year overall survival rates, recurrence rates, operation time, intraoperative blood loss, surgery-related complications, and quality of life.

**Discussion:**

The results of the trial will provide valuable evidence for guiding clinical decision of choosing appropriate treatment strategies for stage IA1 (LVSI) and stage IA2 cervical cancer patients.

**Trial registration:**

ClinicalTrials.gov (NCT04934982, Registered on 22 June 2021).

## Background

Cervical cancer is the most common malignant tumors of female reproductive system in the world [1]. Surgery is the adaptive therapy for early International Federation of Gynecology and Obstetrics (FIGO) stage [including stage IA1 with lymphovascular space invasion (LVSI), IA2, IB1, IB2, IB3, IIA1, and IIA2] cervical cancer [2]. Radical hysterectomy (RH) has been a classic landmark surgery procedure of gynecologic operation methods since 1895. The procedure has gone through several stages of development: the original abdominal RH was conducted by Clark from John Hopkins University in 1895; the “standard” RH was developed by Wertheim from Austria in 1911 and the same year transvaginal RH was performed by Schuchart also from Austria; the “Three fossa theory” RH was proposed by Latzko from Austria in 1919; and the nerve-sparing RH was developed by Okabayashi from Japan in 1952. In 1974, Piver et al. defined the degree of radical resection of the operation and established five types (I-V) of resection standards. Among them, type II surgery is suitable for patients with stage IA1 (LVSI) [3]. In 2008, French gynecologist Querleu and American gynecologist Morrow jointly proposed the milestone Querleu-Morrow classification method (Q-M classification) of radical surgery of cervical cancer. Among them, type B, also called modified radical hysterectomy, is suitable for stage IA1 (LVSI) and IA2 patients [4]. The progress of the masters’ classics has focused primarily on the improvement of the operation procedures.

Modern standard operation procedures include laparoscopic (classified as traditional laparoscopic and robot-assisted laparoscopic surgery, or as porous laparoscopic and single port laparoscopic surgery), abdominal, and transvaginal operation. Since the above mentioned three types of operation procedures have almost the same range of excision using an abdominal, laparoscopic or transvaginal approach, it is intuitively assumed that all three procedures are equally effective. Therefore, most previous studies have focused on the differences in operation time, intraoperative blood loss, hospital stay and cost, but not overall survival (OS) or disease-free survival (DFS). Only several small scale studies have shown that laparoscopic RH was not inferior to abdominal RH in terms of DFS [5–10]. That is why two articles published in the New England Journal of Medicine in late 2018 have shocked the entire gynecology field [11, 12]. The retrospective cohort study concluded that laparoscopic RH was associated with shorter OS than abdominal surgery among patients with stage IA2 and IB1 cervical cancer [11]. Furthermore, the randomized clinical trial (RCT), the Laparoscopic Approach to Cervical Cancer (LACC) trial, revealed that laparoscopic RH was associated with both worse DFS and OS than abdominal RH [12]. Following these two publications, it was recommended that abdominal RH becomes the standard surgery for cervical cancer patients in Version 3.2019 of the National Comprehensive Cancer Network (NCCN) guidelines, which is an enormous change in the field of gynecology.

However, after careful review of the two studies, we have noticed several issues in both studies that cannot be overlooked. The retrospective cohort study could be hampered by significant confounders. There were significant differences of race and socioeconomic status between laparoscopic RH and abdominal RH groups, suggesting possible differences of adjuvant therapy received by the two groups. While in the RCT, the included patients were with stage IA1 (LVSI), stage IA2 and stage IB1 cervical cancer. Among them about 92% were stage IB1 Patients, which should receive subradical or radical hysterectomy according to the staging. No further stratification analysis was performed. In addition, only 10 laparoscopic surgeries were required as the qualification requirement for the surgeon in the trial, which is far from adequate to achieve proficiency at laparoscopic RH.

To address these problems, we designed the current RCT study to compare outcomes of IA1 with LVSI and IA2 stage cervical cancer patients receiving laparoscopic RH vs. abdominal RH under consistent tumor-free operating regulations.

## Methods

### Design

**Fig. 1:**
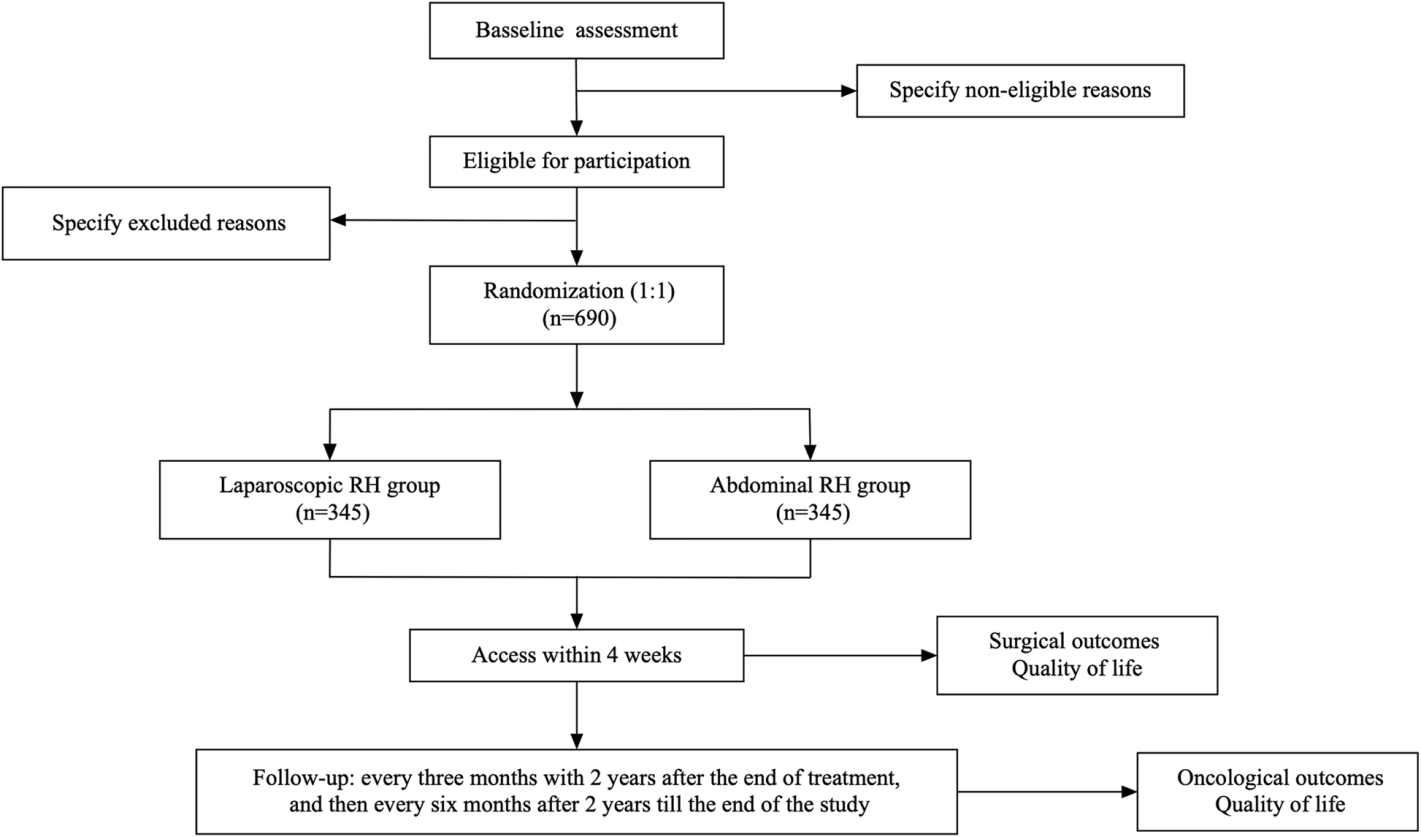
Study flow diagram

This research project is an investigator-initiated, prospective, randomized, open, blinded endpoint (PROBE) non-inferiority controlled trial. Patients are randomly assigned to receive abdominal RH or laparoscopic RH. (Fig. 1). This study protocol adheres to the SPIRIT statement for clinical trial protocols [13] and the SPIRIT-PRO Extension [14]. The study is registered at the ClinicalTrials.gov (NCT04934982, Registered 22 June 2021, https://clinicaltrials.gov/ct2/show/NCT04934982?term=NCT04934982&draw=1&rank=1).

### Participants

Potential participants will be patients with newly diagnosed stage IA1 with LVSI and IA2 cervical cancer at the Obstetrics and Gynecology Hospital of Fudan University, Zhongshan Hospital of Fudan University, Renji Hospital of Shanghai Jiao Tong University, Xinhua Hospital of Shanghai Jiao Tong University, Taizhou Hospital Affiliated Cancer Hospital of the University of Chinese Academy of Sciences, and First Affiliated Hospital of Wenzhou Medical University in China. The staging is confirmed after independent examination of the patients by two senior physicians based on pathology report, as well as pelvic MRI/CT results. Diagnosis of IA1 with LVSI and IA2 requires pathology report of the Loop Electrosurgical Excision Procedure (LEEP). The chief attending physician will enroll the patients.

#### Inclusion criteria

Participants meeting all of the following criteria will be considered for enrollment: (1) clinical diagnosis of squamous carcinoma of the cervix, adenocarcinoma, squamous adenocarcinoma (stage IA1 with LVSI, IA2); (2) age ≥ 21 years and ≤ 70 years; (3) surgery type B (refer to Q-M surgical staging); (4) normal range of liver and kidney function and blood count (hemoglobin > 60 g/L, platelets > 70*10^9^/L, leukocytes > 3*10^9^/L, creatinine < 50 mg/dL, transaminase abnormal indicators ≤ 3, maximum value of transaminases not exceeding 3 times the corresponding normal value); (5) no history of other malignancies; (6) no pregnancy; (7) physical strength classification: Karnofsky score ≥ 60; (8) voluntarily join the study, sign the informed consent form, compliant and cooperative with the follow-up; (9) no psychiatric disorders and other serious immune system disorders (e.g. lupus erythematosus, myasthenia gravis, HIV infection, etc.) (Note: maximum diameter measurement of cervical lesions is based on PET-CT, or CT, or MRI).

#### Exclusion criteria

Participants meeting one of the following criteria will be excluded from enrollment into this study: (1) those who are contraindicated for various surgeries and cannot undergo surgery; (2) patients who have received pelvic/abdominal radiotherapy irradiation or neoadjuvant chemotherapy for cervical cancer; (3) recurrent cervical cancer; (4) patients with CT, MRI or PET-CT suggesting suspicious metastasis of pelvic lymph nodes with maximum diameter > 2 cm after further improvement of preoperative examination.

### Outcome measurements

#### Primary outcome measurements

The primary outcome will be 5 year progression-free survival (PFS), defined according to the response evaluation criteria in solid tumors (RECIST) criteria.

#### Secondary outcome measurements

The secondary outcome measurements will be 5-year overall survival (OS) rates, recurrence rates, operation time, intraoperative blood loss, surgery-related complications, and quality of life.

### Randomization, blinding and treatment allocation

Before randomization, all eligible patients are required to provide written informed consent. A centralized block randomization with a block size of six will be used to randomly assign patients to ensure that there will be equal number of patients (1:1) in the laparoscopic/robotic RH and abdominal RH groups. The randomization scheme will be completed by an independent statistical team at the Clinical Trial Center of the Children's Hospital of Fudan University by creating and sequencing random seed numbers (SAS 9.4 software). The intervention protocols determined by the random assignment sequence will be in sequentially numbered, non-transparent, sealed envelopes by zone: each of the six subgroups for each zone will be placed sequentially in six small, non-transparent, sealed envelopes numbered from 1 to 6, and then uniformly placed in the same large envelope marked with the corresponding zone number. The details of the allocation sequence are unknown to the investigators and surgeons. After completing the baseline measurements, the randomization assignment will be administered by the central coordinator at the Obstetrics and Gynecology Hospital of Fudan University who is not involved in the implementation of the intervention or the outcome observation. The enrollment is competitive, and the sub-center coordinator will contact the central coordinator before the inclusion of the first patient in each group. The central coordinator will follow the randomization scheme and assign the large envelopes with the group numbers to each center following the order of contact. Each sub-center will open the envelopes according to the order of signing the informed consent form, and strictly follow the numbered order of the envelopes, and then inform the participating physicians of the assignment scheme and complete written registration form. The surgeons will perform the operations according to the allocated random number. Treatment allocation will not be blinded to study personnel performing the surgery or patients. Patients will be informed about their group allocation after completion of the pre-surgery evaluation. Research staff evaluating the outcomes will be blinded.

### Accreditation of participating surgeons

Surgeons involved in this project were selected from each participating hospital and are skilled in cervical cancer surgery. To ensure overall quality, the lead surgeon must have experience in at least 50 surgeries each for both LRH and ARH and be able to provide the medical history of these cases. The principal investigator and members of the surgical quality control team will be on site to observe each surgeon's procedure and review the unedited video of the procedure (1 ARH and 1 LRH) provided by each surgeon for surgical quality measurement and documentation to ensure the extent of surgical resection and tumor-free management of each surgeon.

### Surgical treatment

#### General protocol

Minimally invasive radical cervical cancer treatment includes laparoscopic radical cervical cancer treatment (LRH) and robotic-assisted laparoscopic radical cervical cancer treatment, either of which can be chosen by the operator. This project focuses on the survival benefit of patients after minimally invasive and open radical cervical cancer treatment, so radical cervical cancer treatment with preservation of reproductive function will not be included. For both open and minimally invasive radical cervical cancer surgery, the operation begins with a thorough abdominal exploration, including careful exploration of the diaphragm, and any metastatic lesions and metastatic sites should be described in detail in the operative record, with biopsy for confirmative diagnosis. If intra-abdominal lesions are found, radical cervical cancer surgery should not be used and should change to palliative treatment.

Pelvic lymph node dissection should be performed during radical cervical cancer surgery. Sentinel Lymph Node Mapping (SLN) is not included in this project, since there is no sufficient evidence for its sensitivity and specificity, and also the difficulty in achieving lymph node hyperstaging considering the large sample size of this project. If the common iliac lymph node is positive for intraoperative freezing (optional), or if the preoperative evaluation of the paraaortic lymph nodes is positive, paraaortic lymph node dissection is preferred and followed by paraaortic lymph node biopsy. When clear the paraaortic lymph nodes, the upper border reaches the level of the inferior mesenteric artery is enough. The common iliac lymph nodes should be sent separately for examination, and the resection should include both sides of the common iliac vessels, with the upper border reaching the midpoint of the bifurcation of the abdominal aorta and the common iliac vessels and the lower border reaching the level of the bifurcation of the common iliac vessels.

If definite pelvic lymph node metastasis is found intraoperatively, continuation of pelvic lymph node dissection and even radical cervical cancer surgery is not required, but abdominal paraaortic lymph node sampling is recommended to assess the degree of disease progression and to develop subsequent radiotherapy regimens. If surgery is continued, radical cervical cancer surgery + pelvic lymph node dissection + abdominal paraaortic lymph node dissection/biopsy is recommended.

#### Specific operation procedures

1. Open radical hysterectomy for cervical cancer + pelvic lymph node dissection/paraaortic lymph node dissection
  1. The preoperative prophylactic antibiotics are given at least 15 min before incision of the skin.
  2. The sequential compression device (antithrombotic pressure pump) is installed.
  3. The patient is placed in a flat position or bladder lithotomy position.
  4. A median abdominal incision or a low transverse incision is chosen.
  5. After entering the abdomen, a thorough abdominal exploration is first performed, including careful exploration of the diaphragm. Any metastatic lesions and metastatic sites should be described in detail in the surgical record, and biopsy is performed to clarify the diagnosis.
  6. The lateral pelvic peritoneum is opened and any enlarged, suspicious lymph nodes should be removed (if feasible) followed by intraoperative freezing. If these lymph nodes are positive, abdominal paraaortic lymph node biopsy should be performed, but radical cervical cancer surgery may be forgone and the treatment regimen for this subject switched to radical radiotherapy; or radical cervical cancer surgery + pelvic lymph node dissection + abdominal paraaortic lymph node dissection/biopsy may continue.
  7. The radical cervical cancer surgery with or without bilateral salpingo-oophorectomy is performed if there are no suspicious pelvic lymph nodes.
  8. The removal of ovaries: the ovaries may be preserved or removed, or ovarian transposition may be performed, depending on the patient's condition.
  9. The scope of pelvic lymph node dissection: lymph nodes adjacent to the common iliac artery, external iliac artery, internal iliac artery and lymph nodes in the closed fossa should be removed.
  10. The abdominal paraaortic lymph node dissection should reach the level of the inferior mesenteric artery.
  11. Tumor-free principle: use open surgical kidney pedicle forceps to clamp the vagina close, followed by incision of the anterior vaginal wall, insertion of gauze, followed by severance of the posterior wall and disinfection of the vaginal stump.
  12. The abdominal drainage tube is non-compulsory.
  13. The closure of the abdomen layer by layer: closure of the fascia and closure of the skin.
  14. Indwelling urine catheter.
2. Laparoscopic or robot-assisted laparoscopic radical hysterectomy for cervical cancer + pelvic lymph node dissection/paraaortic lymph node dissection.
  1. The preoperative prophylactic antibiotics are given at least 15 min before incision of the skin.
  2. The sequential compression device (antithrombotic pressure pump) is installed.
  3. The patient is placed in bladder lithotomy position with the upper arms flat on the sides of the body or on the chest.
  4. Considering the wide range of surgical instruments used in this type of surgery, the surgeons could choose their preferred laparoscopic instruments.
  5. The approach to the abdomen and the number of puncture points for laparoscopic surgery depend on the operator's personal choice.
  6. After entering the abdomen, a thorough abdominal exploration, including careful exploration of the diaphragm, should be performed first. Any metastatic lesions and metastatic sites should be described in detail in the operative record, and a biopsy should be performed to clarify the diagnosis.
  7. The round ligament is separated in order to open the retroperitoneum. The lateral pelvic peritoneum is opened and any enlarged, suspicious lymph nodes should be resected (if feasible) followed by intraoperative cryopexy. If these lymph nodes are positive, abdominal paraaortic lymph node biopsy should be performed, but radical cervical cancer surgery may be forgone and the treatment plan will be switched to radical radiotherapy in this patient; or radical cervical cancer surgery + pelvic lymph node resection + abdominal paraaortic lymph node dissection/biopsy may be continued.
  8. The radical cervical cancer surgery with or without bilateral salpingo-oophorectomy is performed if there are no suspicious pelvic lymph nodes.
  9. The removal of ovaries: the ovaries may be preserved or removed or ovarian transposition may be performed depending on the patient's specific condition.
  10. The scope of pelvic lymph node resection: the lymph nodes adjacent to the common iliac artery, external iliac artery, internal iliac artery and the lymph nodes in the closed fossa should be removed.
  11. The uterus is not lifted, and methods such as silk traction of the uterine body are used.
  12. The uterine artery is identified and dissociated at the point where the internal iliac artery divides from the uterine artery.
  13. The parametrial tissue and the uterosacral ligament are excised to ensure adequate surgical margins. Pay attention to the tumor- free principle: the vagina is closed before severing it, which can be done by closure of the vagina with an obturator, closure of the vagina with a ligature ring, and closure of the vagina with transvaginal sutures. After laparoscopic closure of the vagina, the vagina is irrigated with sterile water, after which the vagina is severed and the vaginal stump is sutured.
  14. The systematic lymphadenectomy: removal of lymph nodes adjacent to the common iliac artery, external iliac artery, internal iliac artery and lymph nodes in the closed fossa ± parietal aortic lymph nodes (up to the level of the inferior mesenteric artery).
  15. The intraoperative lymph nodes are removed in their entirety, without touching other parts, and placed in a specimen bag for final removal.
  16. The postoperative pelvic and abdominal cavity is suctioned after full flushing with sterile water, and the pelvic flushing fluid is avoided to back up into the upper abdomen during trendelenburg position.
  17. The use of a vaginal apex closure device depends on the individual choice of the surgeon.
  18. The laparoscopic operation can also be done through the vagina.

### Visit procedures

Follow-up will be conducted at the dedicated unit at each center. Written informed consent should be obtained before any protocol-related procedures. See Table 1 for study procedures and baseline and/or follow-up assessments.

**Table 1:** Study procedures and baseline and/or follow-up assessments

Visit Number	Screening for eligibility	Randomization	Blood examination, urinary test, liver and renal function, ECG	Surgical indicators, surgical complications	Gynecological examination	Imaging test	SCCA/CA-125	Cervical liquid-based cytology	HPV test	Adverse events	EORTC QLQ-C30 v3.0; EORTC QLQ-CX24, PRO147(V1.0)
V0	×										
V1		×	×	×	×	×	×	×	×	×	
V2 (3 mos)					×	×	×			×	×
V3 (6 mos)					×	×	×			×	×
V4 (9 mos)					×	×	×			×	×
V5 (1 yr)					×	×	×	×	×	×	×
V6 (15 mos)					×	×	×			×	×
V7 (1.5 yr)					×	×	×			×	×
V8 (21 mos)					×	×	×			×	×
V9 (2 yr)					×	×	×	×	×	×	×
V10 (2.5 yr)					×	×	×			×	×
V11 (3 yr)					×	×	×	×	×	×	×
V12 (3.5 yr)					×	×	×			×	×
V13 (4 yr)					×	×	×	×	×	×	×
V14 (4.5 yr)					×	×	×			×	×
V15 (5 yr)					×	×	×	×	×	×	×

*ECG* Electrocardiogram, *EORTC* European Organization for Research and Treatment of Cancer, *QLQ* Quality of Life Questionnaire, *SCCA* SCC antigen test

At visit 0 (V0) patients are screened for eligibility and eligible patients are scheduled for enrollment.

At the randomization (V1), patients are randomly assigned to either the laparoscopic RH group or the abdominal RH group. Before surgery, the following items will be tested and recorded: routine blood examination, urinary test, liver and renal function, ECG, gynecological examination, SCC antigen test (SCCA) if squamous cell carcinoma/CA-125 if adenocarcinoma, HPV testing, cervical liquidbased cytology, imaging test (chest CT + pelvic enhanced MRI + epigastric enhanced MRI, or PET-CT + pelvic enhanced MRI), the sonographic results (ultrasound of the liver, gall, pancreas, kidney and ureter), and pathology results of tissue biopsy or LEEP results for patents of IA1 with LVSI and IA2 from our hospital. After the surgery, the following items will be tested and recorded: imaging test to evaluate retroperitoneal lymph node metastasis, surgical indicators such as operation time and blood loss, postoperative pain scores and first exhaust time, surgical complications, and adverse events, the period of postoperative fever, the days drainage volume more than 200 mL, and hospital stay.

Clinically follow-up visit will be conducted every three months within 2 years after the surgery, and then every six months after 2 years till the end of the study (Fig. 1). Patients will be sent a text message two weeks before each follow-up visit, and a special made booklet containing follow-up test results will be recorded and kept by the investigators.

At V2-V9 visit, the following evaluation will be performed and results be recorded: imaging test (chest CT + pelvic enhanced MRI + epigastric enhanced MRI, or PET-CT + pelvic enhanced MRI), gynecological examination, SCCA if squamous cell carcinoma/CA-125 if adenocarcinoma, cervical liquid-based cytology, HPV test (only at V3 and V5), adverse events, the European Organization for Research and Treatment of Cancer (EORTC) Quality of Life Questionnaire—Core 30 (QLQ-C30) Version 3.0 to evaluate quality of life, and EORTC Quality of Life Questionnaire Cervical Cancer Module (QLQ-CX24) to evaluate cervical cancer-specific quality of life.

At V10-V15 visit, the following evaluation will be performed and results be recorded: gynecological examination, SCCA if squamous cell carcinoma/CA-125 if adenocarcinoma, cervical liquid based cytology test, high-risk HPV test, imaging test (chest CT + pelvic enhanced MRI + epigastric enhanced MRI, or PET-CT + pelvic enhanced MRI), adverse events, life quality (EORTC QLQ-C30 v3.0), and sex life quality (EORTC QLQ-CX24).

### Description of statistical methods

#### Sample size calculation

This study uses a non-inferiority trial design, and the primary outcome is the 5-year PFS rate. The sample size is calculated based on the difference between the two groups of 5-year PFS rate. The 5-year OS rate of the patients in the open surgery group is estimated to be 96% based on clinical data from our hospital, and the 5-year PFS rate is lacking. To infer the relationship between 5-year PFS rate and 5-year OS rate in patients undergoing open surgery, we refer to previous studies and construct a linear regression equation with 5-year OS rate as the independent variable X and 5-year PFS rate as the dependent variable Y (Y = 1.199 X-0.219). Therefore, the 5-year PFS rate of the open surgery group is estimated to be 93%, when the 5-year OS rate is 96%. Based on the assumptions of (i) a 5-year PFS rate of 93% in the open surgery group, (ii) a non-inferiority margin of 6%, (iii) 80% power, and (iv) a one-sided alpha of 0.025, the sample size estimation resulted in 308 subjects per group (calculated with SAS software, version 9.4). Considering a 10% drop-out rate and the randomization scheme with block size of 6, a total of 690 subjects should be randomized (345 per group).

#### Planned analyses

All analyses will be performed on an intention-to-treat (ITT) basis, except for sensitivity analysis that will be performed according to per-protocol (PP) treatment. All statistical analyses will be performed with a two-sided significance level of 0.05 and conducted using SAS software, version 9.4 (SAS Institute).

1. Analysis of primary outcome data

The curves of PFS at 5 years will be estimated using the Kaplan–Meier method. The logrank test will be used to test the above hypothesis, and the 5-year PFS rate difference and its 95% confidence interval (CI) for the comparison between the two groups will be estimated. The minimally invasive surgery will be considered non-inferior to the open surgery if the one-sided 95% upper limit is less than, where the predetermined non-inferiority margin = 6%. An analysis of the primary outcome adjusting for the blood loss during operation, operative duration, and postoperative pain score will be performed using Cox proportional hazards regression model. The hazard ratio of 5-year PFS and corresponding 95% CI will be estimated. Stratified analysis will be performed according to tumor stage, and Cox proportional hazards regression model will be used to estimate the hazard ratio and corresponding 95% CI of the 5-year PFS.

2. Analysis of secondary outcome data

Continuous outcomes include operation time, anesthesia time, blood loss during operation, postoperative pain score and postoperative hospital stay. The outcomes with normal distribution will be summarized using mean and standard deviation (SD), while the outcomes with non-normal distribution will be summarized using median and interquartile. The differences in the outcomes and 95% CIs will be analyzed by generalized linear model (GLM) with treatment as fixed effect, and with normal distribution and identity link function.

The intraoperative complications, postoperative complications, one-month and one-year postoperative quality of life and sexual life will be treated as binary outcomes and summarized by number (%) of participants with the event. The differences in the outcomes and 95% CIs will be analyzed by GLM with treatment as fixed effect, and with binomial distribution and identity link function.

Adverse events (AEs) will be summarized using the number of AEs, the number (%) of participants with AEs by groups.

### Data confidentiality and storage

The research assistant will collect patient personal information, medical information from medical files after participants’ consent. All data will be quality checked and double entered into a secure electronic database. Paper and electronic data will be stored at designated research office in the hospital, which can only be accessed by research staff. Paper data will be securely held in a locked filling cabinet, and electronic data will be stored on a password-protected computer. Follow-up information will be linked to the baseline clinicopathological database using the unique patient ID number. All identifiable data will be removed for analysis to protect patient’s privacy.

### Ethics and dissemination

The study was approved by the Institutional Review Board of the Obstetrics and Gynecology Hospital of Fudan University in Shanghai, China (Reference number: 2021–03; Date of approval: 18 January 2021). This study is conducted in accordance with the Declaration of Helsinki.

All patients will be given both written and oral information about the study from their gynecologist during the intake for surgery. Patients must sign an informed consent form in accordance with the Declaration of Helsinki before being included in the study. Refusal to participate will not affect standard treatment. Patients can withdraw from the study at any time during the study period. The patients or their family members can address their concerns or queries about the study related questions to the project leaders (HJ or XW) throughout the study period. The investigator has the right to withdraw a patient from the trial treatment or study in the event of secondary disease, adverse events, protocol violations, administrative reasons, or other reasons.

Patients or the public were not involved in the design, or conduct, or reporting, or dissemination plans of this research. An independent data monitoring committee (DMC) composed of a gynecologist, an epidemiologist, and a statistician is installed to oversee patients’ safety and quality of the trial. The DMC met after recruitment had started to establish a charter, and will meet at least annually. Adverse events are recorded and reported by the attending gynecologist according to the charter. The trial steering committee meets every 6 months and will be responsible for overview study progress, drafting annual reports and the final report, and submission for publication. Any modification of the trial protocol will be communicated to relevant parties by the principal investigator after agreement by the steering committee. Study results will be submitted for publication in international medical journals and on the hospital website, and presented at conferences.

## Disscussion

Laparoscopic radical hysterectomy of cervical cancer has been conducted for more than three decades, with continuous improvement of the technique. It has gained popularity by surgeons because of less intraoperative bleeding and faster recovery. However, after the publication of two studies in 2018 [11, 12], NCCN Guidelines version 3.2019 recommends laparotomy as the standard surgery for cervical cancer patients, while laparoscopic surgery is no longer recommended. This is a dramatic change in the field of gynecology.

The LACC trial included patients with stage IA1 (LVSI), stage IA2 and stage IB1 cervical cancer, of which 91.9% were stage IB1 patients. The lesions at stage IA1 (LVSI) and IA2 patients are small and invisible to the naked eye. The surgical method for those patients is type B (modified radical hysterectomy plus bilateral pelvic lymph node dissection, that is, resection of 1 ~ 2 cm parauterine and vagina 1 ~ 2 cm), and paraaortic lymphadenectomy should be performed (based on FIGO 2018 staging and Q-M classification) if necessary. The surgical treatment choice for stage IA1 (LVSI) and IA2 warrants further investigation. Our hospital performs around 1500–2000 cervical cancer surgeries annually over the past 8 years. According to our data, the 5-year OS rate of laparoscopic radical cervical cancer surgery for stage IA1 and stage IA2 from 2014–2018 was 99.49% and 100%, respectively. And the 5-year OS of stage IB1 was 96.93%, which was similar to the open surgery group in the LACC trial. Moreover, our data shows that laparoscopy is superior to open surgery in terms of both short-term complications and long-term survivals for endometrial cancer patients, in which laparoscopic surgery is the first choice recommended by the NCCN guidelines.

Therefore, the current trial aims to specifically focus on stage IA1 (LVSI) and IA2 cervical cancer patients with adequate sample size (n = 690). We enforced a series of stringent criteria to ensure the surgery quality. The participating surgeons need to have the experience of at least 50 operations of LRH and ARH each. The surgical methods are grouped by randomization through the same block to achieve a balance between the two surgical methods for each surgeon. In addition, this trial will strictly follow the above mentioned tumor-free principle as detailed in the Methods section.

So far, there is only one high quality RCT, the LACC trial, comparing the long-term survival of minimally invasive and open radical cervical cancer surgeries. The development and progression of medicine has always been filled with twists and turns. More evidences are needed to guide the clinical decision of choosing the appropriate treatment strategies, as well as improving specific techniques such as for the minimally invasive surgery. Therefore, we designed this study with stringent and consistent standards to compare long-term survivals between LRH (or robot-assisted) and ARH of patients with stage IA1 (LVSI) and stage IA2 cervical cancer.

### Trial status

The study was approved by the Institutional Review Board of the Obstetrics and Gynecology Hospital of Fudan University in Shanghai, China on 18 January 2021. The trial was registered on the ClinicalTrials.gov and the registration number was obtained on 22 June 2021. Recruitment of participants started in June 2021. The last participant is expected to reach the primary endpoint (5-year follow-up) in June 2029. Primary data analysis will begin in June 2026. The protocol version number and date were 1.0 and 21 December 2020, respectively.

## Data Availability

The datasets generated during the current study are available upon request from the principle investigator and meeting the criteria of Shanghai Shenkang Hospital Development Center.

## Abbreviations

DFS: Disease-free survival
DMC: Data monitoring committee
ECG: Electrocardiogram
EORTC: European Organization for Research and Treatment of Cancer
FIGO: International Federation of Gynecology and Obstetrics
HR: Hazard ratios
ITT: Intention-to-treat
LVSI: Lymphovascular space invasion
NCCN: National Comprehensive Cancer Network
OS: Overall survival
PROBE: Prospective, randomized, open, blinded endpoint
PP: Per protocol
PFS: Progression-free survival
QLQ: Quality of Life Questionnaire
Q-M: Querleu-Morrow
RCT: Randomized clinical trial
RH: Radical hysterectomy
SCCA: SCC antigen test
95%CI: 95% Confidence interval
